# Health Tract, No. 223

**Published:** 1865

**Authors:** 


### ELEMENTS OF FOOD.

**The ultimate ingredients of all food are carbon to warm, and nitrogen to make flesh. Some have no carbon, others no nitrogen, some have both in varying proportions, all have water or waste from five to ninety per cent. The table below is the result of the researches of the ablest chemists of the age. The amount of solid matter in an article of food, does not mean that amount of nutriment, for a portion of it may be woody fiber, or waste, or lime, chalk, iron, or other mineral. The cipher indicates that not one per cent of the element is found; n a., not ascertained; blanks, mean no published or reliable statements have been made. The more water, the more waste, for even woody fiber and iron have their essential uses in the system. This and other good tables in this volume should be regarded as merely approximative; they are not so much intended to live by as for guidance in diseased conditions; for example, if constipated, it is better to use rough food, such as has much waste and little nutriment, as fruits, berries, and the like; concentrated food, as boiled rice, is best for loose bowels; syrups, and oils, and milk cause biliousness and fevers; sours, as berries, fruits, and cold slaw, cure fevers. It is safer, however, especially, in health, to eat by instinct rather than by rules or scientific tables.**

| In 100 parts of, there is per centage of | Solid matter. | Water. | Carbon. | Nitrogen. |
|---|---|---|---|---|
| Arabic, gum, | 88 | 12 | 36 | 0 |
| Artichokes, | 28 | 80 | 9 | 0 |
| Apricots, | 25 | 75 | | n.a. |
| Arrow-root, | 82 | 18 | 36 | n.a. |
| Almond oil, | 100 | 0 | 77 | 0 |
| Butter, | 83 | 17 | 66 | n.a. |
| Bread, | 68 | 32 | 31 | n.a. |
| Beans, | 87 | 14 | 38 | n.a. |
| Blood, | 20 | 80 | 10 | 3 |
| Beef, fresh, | 25 | 75 | 10 | 8 |
| Beef tea, | 2 | 98 | — | n.a. |
| Cabbage, | 8 | 92 | — | 0 |
| Carrots, | 12 | 88 | — | 0 |
| Cherries, | 25 | 75 | — | — |
| Cucumbers, | 3 | 97 | — | — |
| Candy, | 90 | 10 | 43 | 0 |
| Egg, white of, | 20 | 80 | — | — |
| Egg yolk, | 46 | 54 | — | — |
| Fish, average, | 20 | 80 | — | — |
| Figs, | 84 | 16 | — | — |
| Gooseberries, | 18 | 81 | — | — |
| Hog's lard, | 100 | 0 | 79 | 0 |
| Isinglass, | 92 | 7 | — | — |
| Leguminous seeds, | 0 | 0 | 37 | — |
| Lentils, | 84 | 16 | 37 | — |
| Manna, | — | 40 | — | — |
| Mutton suet, | 100 | — | 70 | 0 |
| Milk of cow, | 13 | 87 | — | — |
| Milk of ass, | 8 | 92 | — | — |
| Milk of goat, | 13 | 86 | — | — |
| Olive oil, | 100 | — | 77 | — |
| Oats, | 79 | 21 | 40 | 2 |
| Oat meal, | 93 | 7 | — | — |
| Oysters, | 13 | 87 | 36 | — |
| Peas, | 84 | 16 | — | — |
| Potatoes, | 24 | 76 | 11 | — |
| Peaches, | 20 | 80 | — | — |
| Pears, | 16 | 84 | — | — |
| Poultry, | 23 | 77 | — | — |
| Rye, | 83 | 17 | 39 | 2 |
| Sugar, average, | — | — | 42 | 0 |
| Starch average, | 84 | 16 | 36 | 0 |
| Wheat, | 86 | 14 | 39 | 2 |

